# Reflecting on 2025. Anticipating 2026

**DOI:** 10.1093/sleepadvances/zpag005

**Published:** 2026-01-14

**Authors:** Sean P A Drummond

**Affiliations:** School of Psychological Sciences, Monash University, Clayton, Victoria, Australia

I have now completed my first year as Editor-in-Chief of SLEEP Advances. It was certainly everything I thought it would be and more. I now have even more respect for the work Mary Carskadon did as the founding editor of the journal! Given the one-year milestone, I thought it a good opportunity to reflect on 2025 and provide a preview of what we are planning for 2026.

## Article Types

As readers likely know, we introduced or re-emphasized a number of article types last year. Principle among them is Protocol Papers. As I mentioned in my introductory editorial,^1^ we want SLEEP Advances to be the home of Protocol Papers in the sleep and circadian fields. In 2025, we accepted 11 Protocol Papers, including papers from across the globe,^2–5^ systematic review protocols,^5^ mechanism studies,^2^ observational studies,^6^ and clinical trials.^7^ I would love to see that number increase in 2026. To highlight and promote these manuscripts, we will bundle them together in a Special Collection called *Advances in Sleep and Circadian Protocols 2025*. Oxford University Press will promote this Collection to provide maximum exposure for your Protocol Papers. We plan to promote a similar Collection at the start of each year, highlighting the Protocol Papers from the prior year.

In addition to encouraging submissions of Protocol Papers in 2026, SLEEP Advances would like to continue to receive: a) pilot studies and preliminary findings from studies with strong methodologies; b) clinical trials; and c) transfers from SLEEP (which, if transferred after review, typically receive relatively quick decisions).

I would also like to note SLEEP Advances offers flexible format submissions. As long as your submission contains the required elements noted on our Author Guidelines, the exact format for the original submission is flexible. Specific formatting will only be required for revisions.

## Editorial Team

The backbone of any journal is the editorial team. SLEEP Advances has a dedicated and hardworking team.

To acknowledge that dedication and hard work, I am pleased to announce our inaugural class of *Reviewers of the Year!* This is a new initiative recognizing our most outstanding reviewers (among many outstanding reviewers!), based on a combination of the number of reviews completed, the timeliness of the reviews, the quality of the reviews, and Associate Editor recommendations.

The *2025 Reviewers of the Year* are: Chin Moi Chow, Greg Elder, Erin Hanlon, Anthony Reffi, Jennifer Tudor, and Emerson Wickwire. Each receives the heartfelt gratitude of the editors and authors, as well as one publication fee waiver for 2026.

Five Associate Editors ended their terms at the end of 2025. I want to express my deep gratitude to each of them for their thoughtful input, dedication, and tireless efforts on behalf of the journal. They are: Chris Drake, Karen Gamble, Denise O’Driscoll, Bryce Mander, and Jared Saletin.

We also have eight new Associate Editors joining the team in 2026, and I look forward to working with each of them. They we chosen based on a combination of strong performance on the Editorial Advisory Board, significant expertise in areas needed to round out the team, and geographic location. Our new Associate Editors are: Bei Bei (Australia), Emmanuel During (USA), Greg Elder (UK), Jennifer Goldschmied (USA), Anthony Reffi (USA), Catia Reis (Portugal), Amy Reynolds (Australia), and Rebecca Robbins (USA). Dr. Robbins will serve as our Associate Editor for Social Media, helping to boost exposure for the journal and our authors.

In addition to the Associate Editors, several members of the Editorial Advisory Board ended their terms in 2025, and several new members are joining the team in 2026. I thank each member rotating off for their willingness to review and the time and effort they put into providing quality reviews. I welcome the new members, and know they will make strong contributions to the journal.

I also need to thank the Oxford University Press members of our editorial team who work behind the scenes to make SLEEP Advances run well. Without their efforts, the journal would not function. A big thank you to Lake Lloyd, Sara McNamara, and Winnie Titchener.

## Special Collections

In 2025, we finalized or initiated 4 Special Collections. We published Collections on: (1) Genetic and other molecular underpinnings of sleep, sleep disorders, and circadian rhythms including translational approaches (including papers focused on humans,^8^ mice,^9^ and model systems^10^); and (2) Sleep and circadian health in the justice system (including papers on first responders,^11^ those working in the justice system,^12^ those incarcerated,^13^ qualitative studies,^14^ and a clinical trial^15^). We also finalized the Festschrift in honor of Robert Stickgold (including empirical studies,^16^ a review introducing a new theory,^17^ and even a paper from the honoree himself^18^), and published our first papers in the Consumer Sleep Technology Collection.^19–22^ We will formally promote both of these new Collections as soon as the final manuscripts are published.

Looking forward to 2026, we ran a survey asking members of the Sleep Research Society and Australasian Sleep Association to help us determine which Special Collections we should run this year. Thank you to the 120 members who responded! Several of the topics were very popular, suggesting a strong appetite for Special Collections.

## The Three 2026 Special Collections Will Be

Sleep and Circadian Mechanisms in Mental Health.

AI, Machine Learning, and Data Science in Sleep and Circadian Rhythms.

Lived Experience and Codesign in Sleep and Circadian Science.

The first one is set to launch soon, with an anticipated submission deadline in late June 2026. The latter two will be launched later in the year with submission deadlines in the third or fourth Quarter. The exact title and scope for each will be developed by the Guest Editors. If you are potentially interested in serving as a Guest Editor for one of these Collections, please reach out.

In addition to the Special Collections, 2026 will also see a Supplement Issue focused on better understanding sodium oxybate (SO) therapy, with a focus on cardiovascular safety, interpretation of current guidelines, and shared decision-making in patient care.

## Thank You

There is more to say about all we did at SLEEP Advances in 2025 and all we plan for 2026. No one wants to read a long editorial, though. So, I will stop here.

Thank you to everyone who read SLEEP Advances in 2025, everyone who submitted a paper, everyone who reviewed for the journal. In the end, we do this for you and for the field. I look forward to continuing to lead SLEEP Advances in 2026.
